# Textile electromyography electrodes reveal differences in lower limb muscle activation during loaded squats when comparing fixed and free barbell movement paths

**DOI:** 10.3389/fspor.2022.1021323

**Published:** 2022-11-29

**Authors:** Felicia Svensson, Ulrika Aasa, Andrew Strong

**Affiliations:** Department of Community Medicine and Rehabilitation, Physiotherapy, Umeå University, Umeå, Sweden

**Keywords:** electromyography, weightlifting, resistance training, motor control, squat

## Abstract

**Introduction:**

Traditional recordings of muscle activation often involve time-consuming application of surface electrodes affixed to the skin in laboratory environments. The development of textile electromyography (EMG) electrodes now allows fast and unobtrusive assessment of muscle activation in ecologically valid environments. In this study, textile EMG shorts were used to assess whether performing squats with the barbell resting freely on the shoulders or using a Smith machine for a fixed barbell movement path is preferable for maximizing lower limb muscle activation.

**Methods:**

Sixteen athletes performed free and fixed barbell squats in a gym with external loads equivalent to their body mass. Quadriceps, hamstrings and gluteus maximus activation was measured bilaterally with textile EMG electrodes embedded in shorts.

**Results:**

Mean quadriceps activation was greater for the free compared with the fixed movement path for the right (mean difference [MD] 14μV, *p* = 0.04, η*p*^2^ = 0.28) and left leg (MD 15μV, *p* = 0.01, η*p*^2^ = 0.39) over the entire squat and specifically during the first half of the eccentric phase for the left leg (MD 7μV, *p* = 0.04, *d* = 0.56), second half of the eccentric phase for both legs (right leg MD 21μV, *p* = 0.05, *d* = 0.54; left leg MD 23μV, *p* = 0.04, *d* = 0.52) and the first half of the concentric phase for both legs (right leg MD 24μV, *p* = 0.04, *d* = 0.56; left leg MD 15μV, *p* = 0.01, *d* = 0.72). Greater hamstrings activation for the free path was seen for the second half of the eccentric phase (left leg MD 4μV, *p* = 0.03, *d* = 0.58) and first half of the concentric phase (right leg MD 5μV, *p* = 0.02, *d* = 0.72). No significant differences were found for gluteus maximus.

**Discussion:**

Textile EMG electrodes embedded in shorts revealed that to maximize thigh muscle activity during loaded squats, a free barbell movement path is preferable to a fixed barbell movement path.

## Introduction

The loaded barbell squat is a multi-joint exercise for which the prime movers are the quadriceps, hamstrings and gluteals. The squat has neuromuscular as well as biomechanical similarities to jumping and running and is therefore a commonly used exercise to increase muscle strength among athletes across a range of sports (1). Depending on the athletes' goals, the squat can be performed using several alternative techniques, e.g., squatting with the barbell lower down on the back increases hip flexion and reduces knee flexion (2), whereas gluteus maximus activation has been shown to increase when using a wider foot placement (3) and when squatting deeper (>90° knee flexion) (4). Squats can also be performed using different kinds of equipment, such as different footwear (5), unstable surfaces (6), chain-loaded variable resistance (7) or with a Smith machine.

A Smith machine is a stable rack with two parallel tracks which fixate the movement of the barbell in a vertical path. It is often debated whether performing squats with the barbell resting freely on the shoulders or using a Smith machine is preferable for leg muscle strength training. Current evidence indicates that when the squat is performed in a fixed vertical movement path, greater absolute loads can be lifted (1, 8). Schwanbeck et al. observed less gastrocnemius, biceps femoris and vastus medialis activation for Smith machine squats compared with a free movement path among six healthy individuals with strength training experience when using a load equivalent to their eight-repetition maximum (1). When squatting with a lower load (29.5 kg and 60% of body weight), Andersson et al. reported less activation of the soleus muscle, but greater activation of the vastus lateralis and no difference in biceps femoris activation in 14 competitive male athletes when squatting in the Smith machine compared with free weight squats (6). It should however be noted that neither of these studies standardized squat depth or width between feet and nor did they examine muscle activation in the non-dominant side or gluteal muscles (1, 6).

Textile electromyography (EMG) electrodes embedded in clothing allow muscle activation to be recorded in previously inaccessible settings/activities and have been found to be safe to use in human studies (9). From a practical perspective, textile electrodes offer several benefits over traditional surface EMG, such as reduced setup costs, ease-of-use and reduced processing (10). Validity of the signals provided by EMG shorts has been shown to be in good agreement with the traditionally measured surface EMG signals (11). The EMG shorts and traditional surface EMG electrodes have similar within-session repeatability, day-to-day variability as well as muscle strength and EMG relationship (11, 12). The left-right muscle activation ratio in daily activities has also been found to be reliable in healthy individuals (13). Textile electrodes used in EMG shorts can therefore be considered a valid and feasible method for assessing muscle activation (12).

Comparing muscle activity between squats of different movement paths requires standardization of potentially confounding variables that may influence the outcomes of interest. For example, gluteus maximus activation increases when the external load is higher (3), with a wider foot placement (3, 14) and with a depth of ≥90° knee flexion (4). Gluteus medius also seems to reach higher degrees of activation with a wider foot placement (15° hip abduction) (15). Quadriceps activation does not however seem to change due to wider foot placement (14, 16) or different hip joint rotation angles (16). Regarding squat depth, vastus medialis shows lower and gluteus maximus higher percentage contribution in the deep squat compared to parallel or partial squats (2, 4). Further, rectus femoris shows greatest activation between 60 and 90° compared with 0–60° knee flexion (17).

To summarize, the loaded barbell squat is a commonly used exercise to increase thigh and gluteal muscle strength among the general population and athletes. To date, there is a lack of knowledge regarding whether performing squats with the barbell resting freely on the shoulders or using a Smith machine is preferable for increasing lower limb muscle activity. Textile EMG electrodes facilitate the assessment of muscle activation in the field. The aim of the present study was to compare thigh and gluteal muscle activation when performing the loaded barbell squat in a free movement path and in a fixed vertical movement path in healthy athletes under standardized conditions using textile EMG shorts. We hypothesized that the free movement path would result in greater activation of the quadriceps, hamstrings and gluteus maximus compared with the fixed movement path.

## Materials and methods

### Participants

Participants were recruited by contacting and asking coaches of local sports teams to inform their athletes about the study. Eighteen athletes subsequently contacted the study leader (FS) for further information and were screened for the following eligibility criteria: (1) 18 years of age or older, (2) regular use of the squat exercise as part of their strength training during the previous year, (3) healthy and free from pain and/or injury in the back, pelvis or legs for at least the previous 3 months, and (4) full comprehension of written and oral instructions in Swedish and/or English. One individual was excluded because of pain in the back and knees and one was unable to complete data collection. Thus, 16 athletes (ten males, six females) aged 18–31 years (mean age 22.8 ± 4.3 years) with 2–14 years' experience of using the squat as part of their strength training were included. The included participants were track and field runners (*n* = 8), handball (*n* = 4) and soccer (*n* = 3) players, and a swimmer (*n* = 1). Background data for the included participants are presented in Table 1. All participants provided their prior written informed consent. This study was approved by The Swedish Ethical Review Authority (Dnr. 2019-05986). All procedures and ethical principles were in accordance with the Declaration of Helsinki.

**Table 1:** Participant characteristics (*n* = 16; female/male = 10/6).

	**Mean ±SD**	**Minimum**	**Maximum**
Age (years)	22.8 ± 4.3	18	31
Height (m)	1.74 ± 0.06	1.65	1.84
Mass (kg)	67.7 ± 8.9	60.0	90.0
BMI	22.4 ± 2.0	18.5	26.6
**Experience (years)**			
Squats	6.0 ± 3.5	2	14
Strength training	7.1 ± 2.9	4	14
Sports	12.9 ± 5.0	4	24
**Training sessions per week**			
All training	7.1 ± 1.7	5	11
Strength training	2.3 ± 0.8	1	4
Sports	4.8 ± 1.6	2	8
**Training hours per week**			
All training	11.6 ± 4.6	5	24
Strength training	3.4 ± 1.3	1	6
Sports	8.2 ± 3.9	2	18

### Procedures

Data collection was performed in a physiotherapy clinic in the south west of Sweden. The entire testing session was supervised by a physiotherapist (FS) experienced in supervising strength training. Participants first completed a questionnaire to provide background information such as age, sex and training experience. A warm-up was performed which consisted of 15 min of cycling at 60 rpm (self-selected resistance), self-selected flexibility exercises, and one set of ten squats performed with the unloaded 20 kg barbell in a free movement path. Two to three sets of three repetitions of light-load squats (self-selected load) in a free movement path were also performed, with a between-set rest of at least 1 min. The warm-up was also used to practice the standardized squat speed (2 s each for eccentric, concentric, and stand phases), depth (90° knee flexion) and foot placement (shoulder width). Squat speed was standardized using a metronome set at 60 beats per minute and verbal cues as required. Squat depth was standardized by instructing the participants to contact with their posterior thighs a thin rubber band which was stretched across safety bars. A mobile telephone camera was used to record the squats in the sagittal plane to ensure that the desired squat depth had been achieved for each repetition throughout all testing. Foot placement was instructed to be shoulder width. The distance between the medial part of the calcaneus and medial part of the first metatarsophalangeal joint was measured during the warm-up and was applied for all subsequent squats.

### Loaded barbell squats

Five barbell squats were performed for each of two conditions: (1) Free movement path—barbell resting freely on the shoulders, and (2) Fixed vertical movement path—barbell resting on the shoulder as part of a Smith machine. The barbell load for all squats was equivalent to the body mass of each respective participant. This load was chosen to be relatively heavy for the athletes without exposing them to unnecessary risk of injury. The barbell for the free movement path weighed 20 kg and the Smith machine barbell weighed 15 kg. Weight plates were attached to the barbells to reach the required loads, taking into consideration the weight of each respective barbell. Barbell placement for all squats was on the shoulders in a high-bar position (2), i.e., just below the spinous process of the seventh cervical vertebra. The order of condition was pseudo-randomized within each sex.

### Electromyography

**Figure 1:**
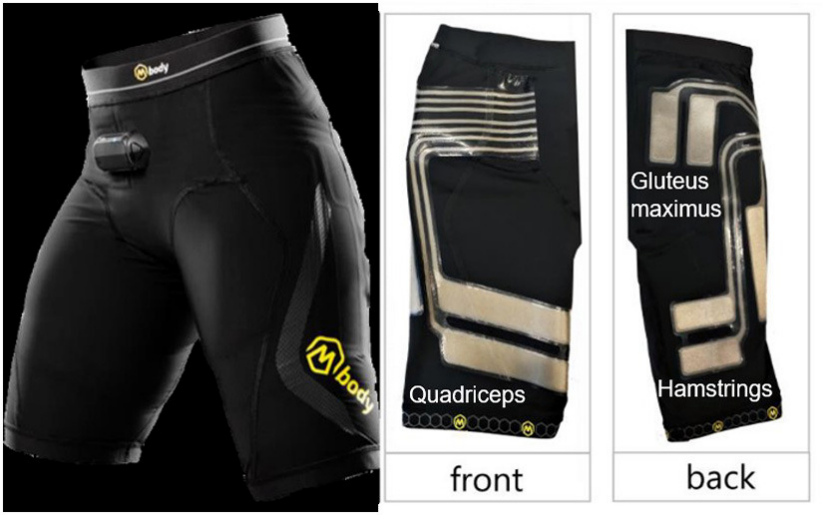
Images of the EMG-embedded shorts Mbody 3, Myontec Ltd., Kuopio, Finland. Front showing the electrodes for quadriceps and the back showing electrodes for hamstrings and gluteus maximus muscles.

Quadriceps, hamstrings and gluteus maximus activity were recorded by EMG-embedded shorts (Mbody 3, Myontec Ltd., Kuopio, Finland, see Figure 1). These muscles were chosen as they are the prime movers of the barbell squat exercise. The EMG shorts were available in three sizes (small, medium, large). Participants tried on the shorts and the most appropriate size was determined as that which did not result in the electrodes moving in relation to the limb, i.e., were too large, and did not limit range of motion, i.e., were too small. The electrodes and wires of the EMG shorts are integrated into the fabric and are thus fixed on the shorts. The electrodes collect data from a larger surface area than traditional electrodes. Activity for the quadriceps and hamstrings thus represented the muscle groups as a whole rather than individual muscles. The electrodes are laminated and consist of conductive silver-coated yarn. The silver fibers typically have an electrical resistance of 10 Ω/10 cm in dry electrodes. Prior to donning the shorts, the electrodes were wetted with water and the skin was prepared with gel to improve signal conduction. The wires were connected to an electronic module, MCell 3, which contained a microprocessor with embedded software, data memory and interface to a computer. A wireless transmitter-receiver enabled signal storage and online monitoring with a computer (12). The raw EMG signals were collected at a sampling frequency of 1,000 Hz. The MCell 3 then further rectified the raw signal, filtered the frequency with a 40–200 Hz band-pass filter and digitalized with a 24-bit A/D converter and a Gain of 0. Prior to warm-up procedures, EMG signals were checked for quality with the participants in relaxed sitting and periodically contracting the muscles of interest. The processed EMG signal was averaged at intervals of 25 samples per second, 25 Hz. The Muscle Monitor Windows software (Myontec Ltd., Kuopio, Finland) was used to analyze the recorded EMG signals.

### Statistical analyses

Of the performed five repetitions for each condition, we included EMG data from only the mid three repetitions in the analyses. The first repetition was excluded because some participants failed to maintain the requested speed and the last repetition was excluded due to visible and/or reported fatigue in some participants. For each muscle group, EMG signals were averaged using the Muscle Monitor Windows software. The software was also used to calculate the mean activation of each muscle group and each repetition for the four following phases: (1) *Initial descent*—the first half of the eccentric phases, (2) *Final descent*—the second half of the eccentric phase, (3) *Initial ascent*—the first half of the concentric phase, and (4) *Final ascent*—the second half of the concentric phase. The start frame of the *Initial descent* phase was determined visually using the analysis software and was defined as the onset of clear muscular activity. End and start frames of the following phases were defined at 1-s intervals thereafter. Mean muscle activation for both legs for each muscle group were compared between the two conditions using a two-way repeated measures ANOVA (main effect within the participants was contrast between conditions and possible interaction effect between muscle activation and order of the two conditions). Partial eta squared (η*p*^2^) was used to estimate effect sizes. Paired samples *t*-tests were used to compare muscle activation between conditions for each defined squat phase. Cohen's d (*d*) was used to estimate effect sizes. Within-session reliability of the EMG data averaged over the entire squat was assessed separately for each muscle group using intraclass correlation coefficient (ICC) estimates and their 95% confidence intervals based on single measures, absolute agreement, two-way mixed effects model in line with recommendations by Trevethan (18). Interpretation of the ICC estimates was made according to Fleiss (19) so that < 0.40 = poor, 0.40–0.75 = fair to good, and >0.75 = excellent. Statistical analyses were performed using the Statistical Package for the Social Sciences (SPSS) analytical version 26 (IBM Corp., Armonk, NY, USA). Statistical significance level for all analyses was set to 0.05.

## Results

### Muscle activation over the entire squat

**Figure 2:**
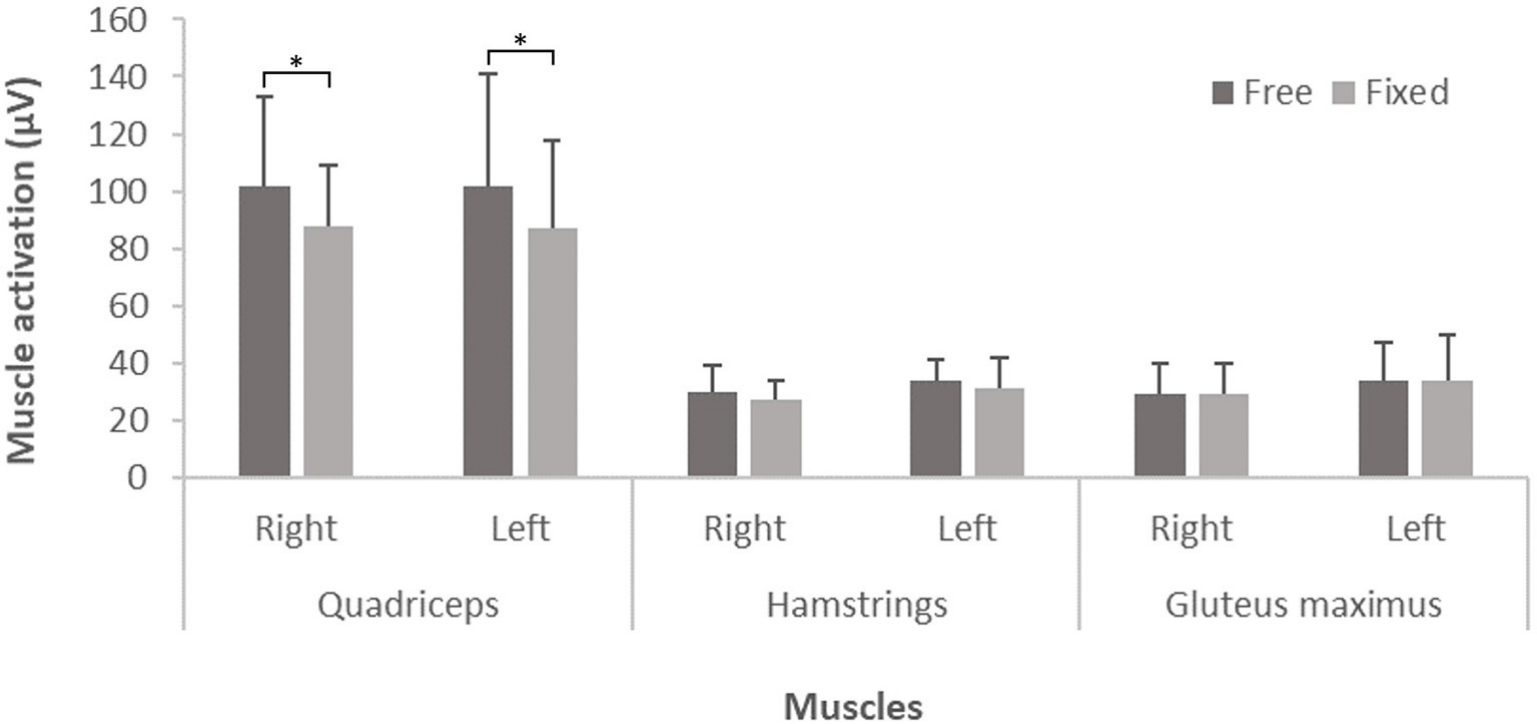
Mean ± standard deviation of muscle activation (min μV) of the entire squat of the mid three repetitions of the loaded barbell squat in quadriceps, hamstrings and gluteus maximus when performed in a free movement path and in a fixed vertical movement path, respectively (*n* = 16). An asterisk indicates a statistically significant difference in muscle activation between conditions. A two-way repeated measures ANOVA was used to compare muscle activation between conditions, i.e., free vs. fixed movement path. The order of the two conditions did not interact with the main effects.

When averaged over the entire squat, quadriceps activation was significantly greater when the loaded barbell squat was performed in a free movement path compared with a fixed movement path for both the right [mean difference (MD) 14 μV, *p* = 0.04, η*p*^2^ = 0.28] and left leg (MD 15 μV, *p* = 0.01, η*p*^2^ = 0.39). Mean activation of the hamstrings and gluteus maximus when averaged over the entire squat was not significantly different between the two conditions. Order of the two conditions was not a significant between-subjects factor in the GLM analyses for any of the analyzed muscles over the entire squat. Mean activation of each muscle for the three repetitions of the entire squat for each condition is illustrated in Figure 2.

### Muscle activation for each squat phase

When averaged for each of the four squat phases, quadriceps activation was significantly higher for the *Final descent* and *Initial ascent* phases when using a free movement path compared with a fixed path for both the right (MD 21 μV, *p* = 0.05, *d* = 0.54 and MD 24 μV, *p* = 0.04, *d* = 0.56, respectively) and left leg (MD 23 μV, *p* = 0.04, *d* = 0.52 and MD 25 μV, *p* = 0.04, *d* = 0.72, respectively). The same was true for the *Initial descent* phase for the left leg (MD 7 μV, *p* = 0.04, *d* = 0.56). Hamstrings activation was significantly greater during *Final descent* for the left leg (MD 4 μV, *p* = 0.03, *d* = 0.58) and *Initial ascent* for the right leg (MD 5 μV, *p* = 0.02, *d* = 0.72) for the free movement path compared with the fixed movement path. There were no differences in gluteus maximus activation between the two conditions for any part of the squat. Order of the two conditions was not a significant between-subjects factor in any of the GLM analyses when muscle activity was averaged for each squat phase. Corresponding mean muscle activation for each squat phase is presented in Table 2.

**Table 2:** Mean ± standard deviation of muscle activation (min μV) for each second of the mid three repetitions of the loaded barbell squat in quadriceps, hamstrings and gluteus maximus when performed in a free movement path and in a fixed vertical movement path, respectively (*n* = 16).

	**Barbell movement path**			
**Muscle group**	**Free**	**Fixed**	***P* ^a^**	***d***
**Quadriceps**
Right leg, Initial descent	62 ± 17	55 ± 21	0.478	–
Left leg, Initial descent	60 ± 22	53 ± 24	**0.042**	**0.56**
Right leg, Final descent	171 ± 58	150 ± 41	**0.050**	**0.54**
Left leg, Final descent	174 ± 80	151 ± 73	**0.035**	**0.52**
Right leg, Initial ascent	136 ± 58	112 ± 43	**0.038**	**0.56**
Left leg, Initial ascent	134 ± 53	109 ± 39	**0.014**	**0.72**
Right leg, Final ascent	40 ± 17	36 ± 10	0.365	–
Left leg, Final ascent	39 ± 15	34 ± 11	0.221	–
**Hamstrings**
Right leg, Initial descent	20 ± 4	20 ± 5	0.740	–
Left leg, Initial descent	26 ± 10	25 ± 14	0.426	–
Right leg, Final descent	34 ± 10	31 ± 11	0.066	–
Left leg, Final descent	37 ± 8	33 ± 6	**0.033**	**0.58**
Right leg, Initial ascent	38 ± 13	33 ± 10	**0.015**	**0.72**
Left leg, Initial ascent	42 ± 10	36 ± 11	0.066	–
Right leg, Final ascent	26 ± 14	22 ± 12	0.313	–
Left leg, Final ascent	31 ± 10	30 ± 21	0.822	–
**Gluteus maximus**
Right leg, Initial descent	18 ± 10	19 ± 10	0.800	–
Left leg, Initial descent	22 ± 13	22 ± 12	0.872	–
Right leg, Final descent	25 ± 9	26 ± 9	0.808	–
Left leg, Final descent	29 ± 12	28 ± 10	0.667	–
Right leg, Initial ascent	39 ± 14	37 ± 13	0.561	–
Left leg, Initial ascent	45 ± 23	44 ± 23	1.000	–
Right leg, Final ascent	34 ± 21	33 ± 19	0.292	–
Left leg, Final ascent	40 ± 22	42 ± 29	0.634	–

^a^Paired samples *t*-tests were used to compare muscle activation between conditions.

Bold *p*-values indicate significant differences. Cohen's d (*d*) is provided as an estimate of effect size for all significant results.

### Reliability of EMG data

For within-session reliability of the EMG data, ICC ranged from 0.85 to 0.96 (95% CI 0.69–0.99) (see Table 3) and was thus interpreted as excellent for all muscle groups.

**Table 3:** Within-session reliability of the EMG data for both squat conditions (free or fixed barbell movement path), all muscle groups and each leg.

**Muscle group**	**Free**	**Fixed**
**Quadriceps**
Right leg	0.89 (0.76–0.96)	0.85 (0.69–0.94)
Left leg	0.93 (0.85–0.97)	0.93 (0.86–0.97)
**Hamstrings**
Right leg	0.95 (0.89–0.98)	0.89 (0.77–0.96)
Left leg	0.90 (0.78–0.96)	0.95 (0.88–0.98)
**Gluteus maximus**
Right leg	0.96 (0.92–0.99)	0.95 (0.89–0.98)
Left leg	0.93 (0.86–0.97)	0.91 (0.79–0.97)

Intraclass correlation coefficients (95% CI) based on single measures, absolute agreement and two-way mixed effects model.

## Discussion

Performing squats with a free barbell movement path produced significantly greater quadriceps muscle activation compared to performing squats with a fixed barbell movement path in a Smith machine. This difference in quadriceps activation was most prominent during the *Final descent* phase (i.e., the second half of the eccentric phase) and *Initial ascent* phase (i.e., first half of the concentric phase). These phases of the squat are when the knees are most flexed and subsequently when the quadriceps display peak EMG activity (20). Although hamstrings activation was not significantly different between conditions when averaged over the entire squat, greater activation was seen during the *Final descent* and *Initial ascent* phases for the right and left leg, respectively. No difference between conditions was evident for the gluteus maximus in any of the analyses. These results support common beliefs that squats performed in a free movement path may activate some muscles to a higher extent than when performed in a fixed vertical movement path.

Our findings of greater quadriceps muscle activation for a free barbell movement path are consistent with the results shown in the study by Schwanbeck et al. (1), whose participants performed eight consecutive squats with a load equivalent to their eight-repetition maximum. Conversely, Anderson and Behm (6) found greater activation of the quadriceps when squatting in the Smith machine compared to squatting with a free barbell movement path when performing only one repetition with three different loads; no resistance, the weight of the Smith machine barbell (29.5 kg) and a load corresponding to 60% body mass. A possible explanation for this discrepancy could be differences in foot placement, where in our protocol the feet were positioned directly under the barbell, whereas in the study by Anderson and Behm, participants appear to have performed the Smith machine squat with their feet anterior to the barbell. The anterior position of the feet to the barbell allows lifters to push backwards into the Smith machine and subsequently increase stability and activation of the vastus lateralis (6).

Quadriceps activation was significantly greater for the free movement path compared with the fixed movement path when the knee and hip joints were more flexed. One reason for this could be that apart from being the primary muscles used to extend the knee in the ascending phase (quadriceps) and to act as a synergist to support the gluteus maximus in hip extension (hamstrings), parts of these muscles also act as stabilizers to support the knee joint when it is flexed. For example, when the knees are in the greatest flexion at the bottom of the squat, hamstrings activation helps to stabilize the knee joint by countering the forces of the quadriceps to extend the leg. The need for co-contraction of the agonist and antagonist may therefore play a particularly important role when performing the squat in a free movement path (1). Another reason may be that the same absolute load was used for both conditions for standardization purposes. Previous research has found that performing squats in a Smith machine results in greater one repetition maximum (1RM) loads compared to free weight squats (8). This may subsequently lead to a lower relative load in relation to 1RM when squatting in the Smith machine with the same absolute weight as with a barbell resting freely on the shoulders, and therefore lower muscle activation. It is thus possible that differences in loads relative to 1RM led to different results between conditions in our study. Schwanbeck et al. (1) however found similar results to our study when using the same relative loads between conditions.

Gluteus maximus activation was similar when squatting with a free barbell movement path and in a Smith machine. During the squat, the hips move behind the center of mass during the concentric phase. During the eccentric phase, the hips rise up and forward to return in line with the center of mass. The gluteus maximus serves an important function in the squat to bring the hips back into full extension, but does not seem to be influenced by the type of movement path when the foot placement and load is the same. To increase its activation, the gluteus maximus requires greater external load (16), a wider foot placement and/or a greater squat depth than a parallel squat (4, 16).

### Methodological considerations

Muscle activation increases due to higher external loads (16) and it was therefore considered important to standardize this for all participants. We chose a load corresponding to each individual's body mass. The applied load was thus not equivalent in terms of its relationship to each individual's 1RM and thus represents differing levels of effort within the group. It has previously been suggested to use a load based on percentage of 1RM (16, 21). Comparisons were nonetheless not made between participants, but instead between the two different squat conditions within each participant. Thus, the chosen external load is not believed to have influenced the results of this study. We also considered testing for 1RM to be inappropriate as it would have increased the risk for injury when performing maximum repetitions. Notably, other studies have also based the load on the body mass of the participant (4, 22). For example, Caterisano et al. (4) compared muscle activation during three repetitions of three different squatting depths using 100–125% of body mass. The potential influence of friction from the Smith machine was not considered in our study. Such friction is however considered minimal and was believed unlikely to influence our results (8). Further research is also needed to investigate whether altering stance (e.g., positioning of the feet) and squatting depths influences muscle activation between the two barbell conditions.

To measure muscle activity, we used EMG-embedded shorts rather than traditional EMG equipment such as individual surface or fine-wire electrodes. This was partly because our aim was to compare activation of the superficial quadriceps and hamstrings muscle groups, rather than individual muscles, as well as the gluteus maximus. The EMG-embedded shorts thus allowed us to achieve this while also reducing preparation time and increasing comfort for the participants compared with traditional methods. Our study therefore demonstrates that EMG shorts provide a convenient assessment of lower limb muscle activity outside of a laboratory environment. This is valuable evidence for sports coaches, clinicians and individuals with an interest in such analyses. Our findings may help to encourage the use of EMG outside of research settings and facilitate greater insights into motor control across a broader range of individuals. Regarding validity of the signals, EMG shorts have been shown to be in good agreement with the traditionally measured surface EMG signals (11). Both the EMG shorts and the traditional surface EMG electrodes have similar within-session repeatability, day-to-day variability, as well as muscle strength and EMG relationship (11, 12). The left-right muscle activation ratio in daily activities has also been found to be reliable in healthy individuals (13). Thereby, textile electrodes used in such EMG shorts can be considered a valid and feasible method for assessing muscle activation (12). Textile electrodes have also been proven to be safe to use in human studies (9).

### Conclusions

Textile EMG shorts revealed that quadriceps and hamstrings activation is greater when loaded squats are performed with a free barbell movement path compared with a fixed movement path in a Smith machine, but no significant differences were found for gluteus maximus activation. When using a barbell load equivalent to body mass, a free movement path appears preferable to a fixed movement path in a Smith machine when the goal is to maximize lower limb muscle activity.

## Data availability statement

The raw data supporting the conclusions of this article will be made available by the authors, without undue reservation.

## Ethics statement

The studies involving human participants were reviewed and approved by the Swedish Ethical Review Authority. The patients/participants provided their written informed consent to participate in this study.

## Author contributions

UA had the original idea. FS recruited participants and performed data collection and wrote the first draft of the manuscript. UA and FS processed and analyzed the electromyographic data. AS advised on statistical analyses and wrote sections of the manuscript. All authors interpreted the data, read, edited, and approved the final draft of the manuscript.

## Conflict of interest

The authors declare that the research was conducted in the absence of any commercial or financial relationships that could be construed as a potential conflict of interest.

## Publisher's note

All claims expressed in this article are solely those of the authors and do not necessarily represent those of their affiliated organizations, or those of the publisher, the editors and the reviewers. Any product that may be evaluated in this article, or claim that may be made by its manufacturer, is not guaranteed or endorsed by the publisher.
